# altAFplotter: a web app for reliable UPD detection in NGS diagnostics

**DOI:** 10.1186/s12859-024-05922-3

**Published:** 2024-09-12

**Authors:** Maximilian Radtke, Johanna Moch, Julia Hentschel, Isabell Schumann

**Affiliations:** 1 Institute of Human Genetics, Medical Facility, Leipzig University, Leipzig, Germany; 2 Centre of Medical Genetics, Department of Medical Genetics, University of Münster, Münster, Germany

**Keywords:** UPD, UPD-detection, AltAFplotter, NGS-diagnostics, Isodisomy, Heterodisomy

## Abstract

**Background:**

The detection of uniparental disomies (the inheritance of both chromosome homologues from a single parent, UPDs) is not part of most standard or commercial NGS-pipelines in human genetics and thus a common gap in NGS diagnostics. To address this we developed a tool for UPD-detection based on panel or exome data which is easy to use and publicly available.

**Results:**

The app is freely available at https://altafplotter.uni-leipzig.de/ and implemented in Python, using the Streamlit framework for data science web apps. It utilizes bcftools and tabix for processing vcf files. The source code is available at https://github.com/HUGLeipzig/altafplotter and can be used to host your own instance of the tool.

**Conclusion:**

We believe the app to be a great benefit for research and diagnostic labs, which struggle identifying and interpreting UPDs in their NGS diagnostic setup. The information provided allows a quick interpretation of the results and thus is suitable for usage in a high throughput manner by clinicians and biologists.

**Supplementary Information:**

The online version contains supplementary material available at 10.1186/s12859-024-05922-3.

## Background

Despite the rapid advancements in next generation sequencing techniques and continously reducing costs, the lack of bioinformatic expertise in smaller labs prevents the application of certain diagnostic methods. These include the detection of uniparental disomies which are of high clinical relevance [1] and can be identified in large panel, exome or genome sequencing.

In order to enable clinicians and researchers to perform such additional diagnostic steps, we aim to provide a curated application that is easy to use and provides a guideline for interpretation of complex genotypes. The web app "altAFplotter" (https://altafplotter.uni-leipzig.de/) performs a set of analyses and summarizes the results in an overview table but also allows detailed visual exploration of alternative allele frequencies, inheritance patterns and runs of homozygosity (ROH) in an interactive user interface.The app is designed in a way that it can be easily hosted locally and integrated in an existing workflow, as it processes standard vcf files.

## Implementation

The app was designed for usage by staff not trained in bioinformatics. Therefor easy to use upload options and optional integration with external APIs or datasources is possible. Handling of UI, files and visualization is implemented with python and streamlit (https://github.com/streamlit/streamlit) as an web application framework.

For processing of vcf files, we utilize bcftools isec [2] and bcftools roh [3], two widely used and well maintained tools for analysis of vcf files.

## Results

The identification of UPDs and their classification as isodisomy, heterodisomy, mixed or segmental iso-and heterodisomy can be achieved by examination of ROHs and inheritance patterns per chromosome. A batch evaluation of positive controls from previously described cases [4] and our patient cohort (largely western europeans) of ca. 9000 large panel and exome sequencing samples [5] was used to determine cutoffs for chromosome flagging. These samples have been mostly processed according to GATK best practices at the time. Cutoffs for whole genome sequencing data might differ and will be adjusted in a future version.

**Fig. 1:**
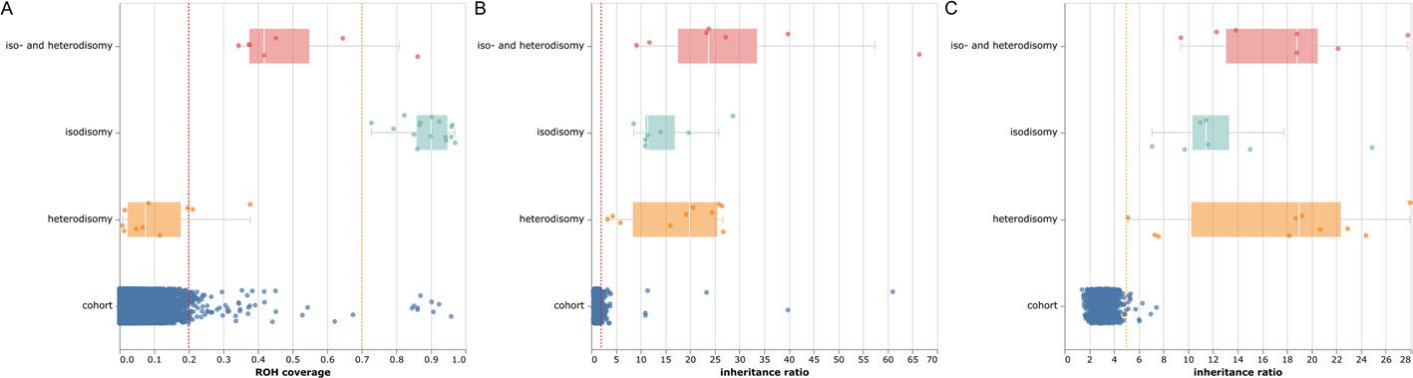
Determination of cutoffs, each data point represents the respective metric (ROH coverage or inheritance ratio) on one chromosome.  "Cohort" refers to an evaluation of ~  6200 whole exomes (a subset of the entire cohort: only whole exomes, no consanguinous patients), "iso- and heterodisomy", "isodisomy" and "heterodisomy" refers to positive controls used to defined cutoffs. **A** ROH coverage cutoffs as defined for isodisomies (0.7, orange line) and mixed UPDs (0.2, red line). Heterodisomies can not be identified based on ROHs. **B** inheritance ratios for trio analyses, shown here is the ratio of maternal over paternal variants. The cutoff (2, red line) includes all positive controls and can be used to identify all three types of UPDs. **C** For duo-analyses, the ratio of maternal over non-maternal or paternal over non-paternal variants can be used to identify UPDs. The cutoff (5, orange line) is chosen to include all positive controls

The cutoffs for flagging were selected to ensure highly sensitive detection (27/27 positive controls are detected, see Fig. 1A–C) at the cost of increased false positives (2% in our exome cohort analysis, excluding consanguinous individuals). As the tool is designed for case by case evaluation with manual inspection, we reason that a high sensitivity is the appropriate approach for a diagnostic setting. For the same reason, we recommend using unfiltered or very moderately quality filtered vcfs. Too stringent quality filtering can favor homozygous variants, which would decrease the sensitivity for ROH-detection. The cutoffs for consanguinity detection are also based on our cohort and were chosen to separate those families for which consanguinity was reported.

### Chromosome flagging is informed by the applied method, ROH detection and inheritance ratio

- Runs of homozygosity: flags are applied, if the chromosome is covered by > 70% (**roh_high**) or between 20 and 70% (**roh_mixed**) ROHs. Applicable for both, single and multi-sample analyses.
- Inheritance ratio: this value describes the ratio between maternal and paternal variants and vice versa in trio setups. For duos (index and one parent) it describes the ratio between maternal and not-maternal or paternal and not-paternal variants. Cutoffs here were chosen as follows: >2 for trios and > 5 for duos. For these chromosomes the flag **inh_ratio_high** is applied. This allows the detection of iso- and heterodisomies and the identification of the parental origin.
- Consanguinity: if more than three chromosomes exceed a ROH coverage of 10% per patient, the flag **consanguinity_likely** is applied. Such cases can not be reliably evaluated by this approach and require carefull manual inspection.
- Insufficient SNVs: if per chromosome less than 200 SNVs are present, the chromosome is excluded from analysis and the flag **insufficient_snvs** is applied.

For interpretation of chromosome flags and their various combinations, Table 1 can be consulted. Large deletions also lead to longer ROHs, therefore an additional hint is given to the user to check for those, if a ROH-flag is applied. For this reason and as a general recommendation, validation of UPDs by a second method is strongly advised. Besides the flagging, detailed and interactive plots are available to allow for investigation of affected chromosomes (see Figure S1).

**Table 1:** Interpretation guidelines for chromosome specific UPD-flags as shown in the web-app

roh_high	Potential isodisomy—check for deletions
roh_mixed	Potential (mixed) isodisomy - check for deletions
inh_ratio_high	Potential isodisomy or heterodisomy, check ROH-flags
roh_high(_mixed) + inh_ratio_high	Potential (mixed) isodisomy

There are some limitations for the analysis of vcf files:

- Size: in the current iteration, we limit the size of vcf files to 200 MB/file to ensure rapid on demand processing. This limits the usage of unfiltered whole genome sequencing data. Future versions will support slimmer data formats such as .baf-files.
- Panels: ROHs tend to be overestimated, if the number of variants is too low. We found 200 variants per chromosome to allow reliable detection of real isodisomies and thus disallow the analysis of chromosomes with less than 200 SNVs to prevent ROH-calling artefacts. Therefore Panels analysed must have a minimum size to allow reliable UPD detection.

## Conclusion

As an easy to use and easy to set up tool, the altAFplotter has proven valuable in the increase of the diagnostic yield in NGS analyses. It allows a quick, yet sensitive evaluation on a case by case basis, that complements and integrates easy with common NGS workflows.

## Availability and requirements

The app is freely available under https://github.com/HUGLeipzig/altafplotter and publicly hosted in the University Leipzig Computing Center under https://altafplotter.uni-leipzig.de/.

As altAFplotter is part of our internal diagnostic pipeline, and we perform regular updates and addition of new features. At the time of writing, next upcoming changes include: support for ROH calling with a public variant reference (e.g. gnomAD) for more relevant ROH detection; improved detection of consanguinity; increased sensitivity for duo setups, where the parent of origin is not sequenced; better integration of whole genome data (such as support for DRAGEN’s BAF-files) and continous improvements in stability and robustness.

User guidelines for general handling and interpretation of the results can be found here: https://github.com/HUGLeipzig/altafplotter/blob/main/user_guideline/user_guideline.md.

Project name: altafplotter.

Project home page: https://github.com/HUGLeipzig/altafplotter.

Operating system(s): Linux.

Programming language: python.

Other requirements: see https://github.com/HUGLeipzig/altafplotter/blob/main/requirements.txt.

License: GNU GPL.

Any restrictions to use by non-academics: see licence.

## Supplementary Information

Supplementary Material 1.

## Data Availability

No datasets were generated or analysed during the current study.

## Abbreviations

altAF: Alternative allele fraction
IR: Inheritance ratio
ROH: Runs of homozygosity
UPD: Uiparental disomy
