# Supplementary figures and images for: altAFplotter: a web app for reliable UPD detection in NGS diagnostics
Source: BMC Bioinformatics. 2024 Sep 12;25:299. doi: 10.1186/s12859-024-05922-3 (PMC11391601; doi:10.1186/s12859-024-05922-3)

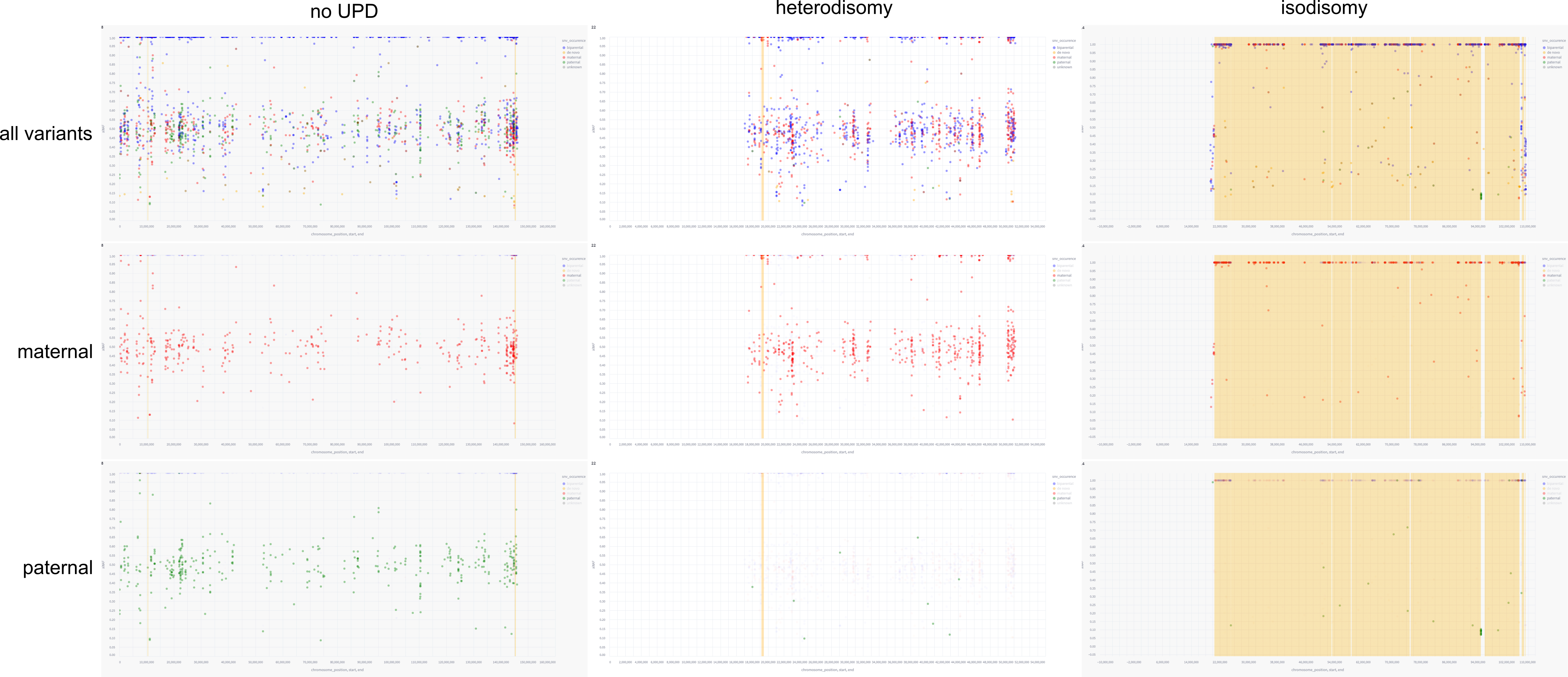

Supplement: Supplementary file 1 — Supplementary Material 1. [file 12859_2024_5922_MOESM1_ESM.png]
